# Cell death and cell lysis are separable events during pyroptosis

**DOI:** 10.1038/cddiscovery.2017.70

**Published:** 2017-11-13

**Authors:** Lucian DiPeso, Daisy X Ji, Russell E Vance, Jordan V Price

**Affiliations:** 1 Division of Immunology & Pathogenesis, Department of Molecular & Cell Biology, and Cancer Research Laboratory, University of California, Berkeley, CA, USA; 2 Howard Hughes Medical Institute, University of California, Berkeley, CA, USA; 3 Department of Biology, Oberlin College, Oberlin, OH, USA

## Abstract

Although much insight has been gained into the mechanisms by which activation of the inflammasome can trigger pyroptosis in mammalian cells, the precise kinetics of the end stages of pyroptosis have not been well characterized. Using time-lapse fluorescent imaging to analyze the kinetics of pyroptosis in individual murine macrophages, we observed distinct stages of cell death and cell lysis. Our data demonstrate that cell membrane permeability resulting from gasdermin D pore formation is coincident with the cessation of cell movement, loss of mitochondrial activity, and cell swelling, events that can be uncoupled from cell lysis. We propose a model of pyroptosis in which cell death can occur independently of cell lysis. The uncoupling of cell death from cell lysis may allow for better control of cytosolic contents upon activation of the inflammasome.

## Introduction

Pyroptosis is a form of pro-inflammatory programmed cell death in mammalian cells that is triggered by activation of various inflammasome complexes, leading to the activation of the proteolytic enzymes caspase-1 or caspase-11 (or caspases 4/5 in humans).^1,2^ In 2015, several groups determined that the pore-forming protein gasdermin D (GSDMD) is cleaved by these pro-inflammatory caspases and is required for cell death during pyroptosis.^3–5^ GSDMD is part of a larger family of gasdermin proteins that share the ability to disrupt cellular membranes upon activation.^6^ In mouse and human cells, pro-inflammatory caspases cleave an autoinhibitory C-terminal domain from the N-terminal domain of GSDMD, which then oligomerizes to form 10–15 nm diameter pores in the cell membrane.^7,8^ GSDMD pores are large enough to allow the release of pro-inflammatory cytokines, IL-1*β* and IL-18, along with an influx of cationic species, notably Ca^2+^, collapsing osmotic and electrical gradients and increasing the tonicity of the cell.^9^ Water influx follows to relieve the osmotic gradient, and in the cell culture conditions under which pyroptosis is normally studied, the cell swells and lyses. Pyroptosis is often measured using an assay to detect the release of the large cytosolic tetrameric complex lactate dehydrogenase (LDH) into the culture media. In this way, LDH release, an indicator of cell lysis, is often interpreted as a measure of cell death, leading many in the field to equate cell death with cell lysis. Pyroptosis has therefore been described canonically as a lytic form of programmed cell death.^1,2,6^ Prevention of cell lysis during pyroptosis using various anti-lytic reagents such as glycine has been suggested to preserve the viability of pyroptotic cells; however, the relationship between cell lysis and cell death during pyroptosis remains unclear.^7,10^

Although inflammasome activation and pyroptosis are often studied in mouse bone marrow-derived macrophages, several studies have reported that other cell types, including neutrophils, fibroblasts, and human monocytes, can undergo inflammasome activation and release smaller proteins (for example, processed IL-1*β*) without evidence of overt cellular lysis (for example, release of LDH).^11–14^ In addition, Jorgensen *et al.*^15^ have recently proposed that pyroptotic cells do not completely disgorge their internal contents into the external environment, but can function as traps termed pore-induced intracellular traps (PITs) to ensnare intracellular pathogens in a digestible carcass, facilitating pathogen clearance by other phagocytes. These observations suggest that the relationships between inflammasome activation, cell death, and cell lysis that occur during pyroptosis may be more nuanced than generally appreciated. Accordingly, it is important to acquire a more precise understanding of the kinetics of cell death *versus* cell lysis that occur during pyroptosis.

To study pyroptosis in the laboratory, we use an inducer of pyroptosis called RodTox. RodTox is a combination of two recombinant proteins: (1) protective antigen (PA) from *B. anthracis*, and (2) recombinant inner rod protein PrgJ from the *S.* *typhimurium* SPI-1 type III secretion system fused to the N-terminal domain of anthrax lethal factor (LFn-PrgJ).^16^ RodTox activates the NAIP2/NLRC4 inflammasome, leading to caspase-1 activation and pyroptosis.^16^ We developed a computational workflow to compile multiple readouts of cell viability and lysis during pyroptosis in individual bone marrow-derived macrophages (BMMs) acquired using time-lapse fluorescence microscopy. Our results revealed distinct stages of cell death and lysis of BMMs following exposure to RodTox *in vitro*. First, cell movement and mitochondrial activity cease coincident with GSDMD pore-induced cell membrane permeabilization and the initiation of cell swelling. In this study, we interpret loss of cell movement and loss of mitochondrial membrane potential as cell death, and we observed that even in normal tissue culture conditions, death precedes cell lysis. Using the anti-lytic reagent glycine, we suppressed cell lysis in pyroptotic cells and found that loss of cell movement, loss of mitochondrial activity, and cell swelling still occur, further suggesting that pyroptotic cell death occurs independently of and prior to cell lysis.

Our results illustrate that although cell lysis can occur following pyroptotic stimulus, lysis is not required for cell death and may not occur in all instances of pyroptosis. Thus, in our study we consider pyroptosis to be gasdermin-dependent cell death that may or may not be associated with cell lysis. A more nuanced understanding of the kinetics of cell death and cell lysis that occur following pyroptotic stimulus may be relevant for several cell types that have been found to undergo inflammasome activation without overt cell lysis or LDH release.

## Results

### Cell lysis can be delayed but not prevented following inflammasome activation

**Figure 1:**
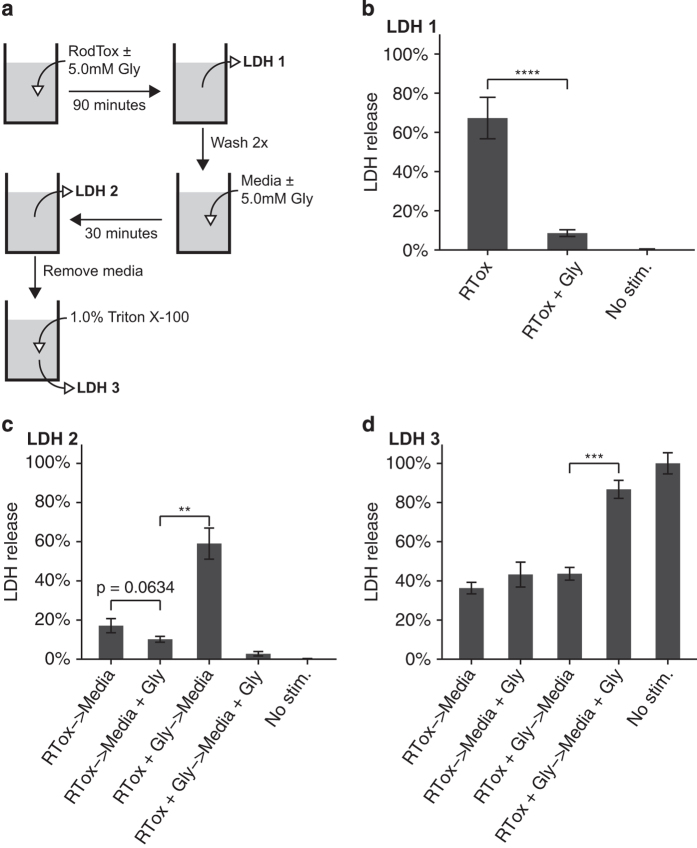
Cell lysis can be delayed but not prevented following pyroptosis. (**a**) Overview of sequential stimulation and LDH measurement. (**b**) LDH release from BMMs treated with RodTox (RTox), RodTox+5 mM glycine (RTox+Gly), or media alone (no stim.) for 90 min. (**c**) LDH release from BMMs in **b** following wash and replacement with media±5 mM glycine for 30 min. (**d**) LDH release from BMMs in **c** following replacement with a 1% Triton X-100 solution in PBS. Data are depicted as % of LDH release from 1% Triton X-100-treated unstimulated BMMs at each step and are representative of three independent experiments. **P<0.01, ***P<0.001, ****P<0.0001, two-tailed Student’s *t*-test. RTox, RodTox; Gly, glycine.

To investigate whether blocking cell lysis can prevent cell death, we performed sequential treatment of BMMs with RodTox±5 mM glycine (Figure 1a). As several other groups have reported, we observed that adding the anti-lytic agent glycine nearly completely blocks LDH release following pyroptotic stimulus of BMMs (Figure 1b).^17–19^ After 90 min, we removed the media from the prior stimulation conditions and replaced them with fresh media±glycine. When glycine was removed, we observed robust LDH release from BMMs previously exposed to RodTox in the presence of glycine (Figure 1c). We observed negligible further LDH release from cells first treated with RodTox alone, regardless of whether replacement media was±glycine (Figure 1c). A final replacement with a 1% Triton X-100 solution to lyse all remaining cells revealed that the proportion of BMMs that remained unlysed was equivalent in all stimulation conditions (Figure 1d). In sum, although LDH release could be delayed by the presence of glycine, once glycine was removed, we observed robust LDH release from all cell populations stimulated with RodTox.

To determine whether longer incubation in glycine would allow BMMs to recover membrane integrity following pyroptosis, similar to a previous observation involving the treatment of bovine vascular endothelial cells with maitotoxin, we exposed BMMs to RodTox for 5 h in the presence of 5 mM glycine.^20^ BMMs treated with glycine for this extended period of time were unable to restore cell membrane integrity following RodTox (Supplementary Figure S1). These results suggest that suppressing lysis does not preserve macrophage viability following treatment with RodTox. Alternatively, the presence of glycine could block the effects of RodTox on treated cells, resulting in delayed inflammasome activation.

### Single-cell live imaging reveals discrete stages of cell membrane permeability and cell lysis following pyroptotic stimulus

**Figure 2:**
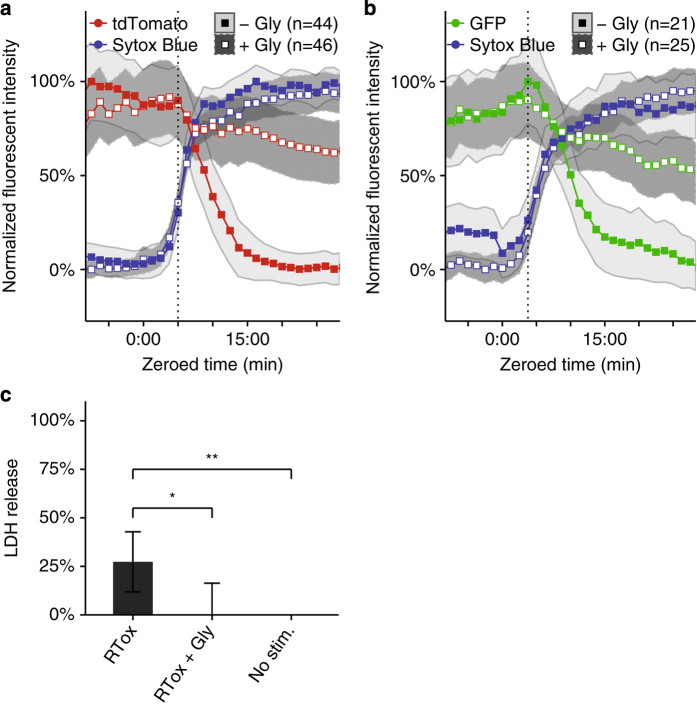
Glycine inhibits cell lysis without affecting plasma membrane permeability. (**a**) Fluorescent intensities over time of Sytox Blue and tdTomato in tdTomato-expressing BMMs treated with RodTox±5 mM glycine. The start of tdTomato loss in the no-glycine condition is annotated with a vertical dotted line. Loss of tdTomato fluorescence is statistically significant±5 mM glycine (rANOVA, *P*=0.00391) (**b**) Fluorescent intensities over time of Sytox Blue and GFP in GFP-expressing BMMs treated with RodTox±5 mM glycine. The start of GFP loss in the no-glycine condition is annotated with a vertical dotted line. Loss of GFP fluorescence is statistically significant±5 mM glycine (rANOVA, *P*=0.0442). Data in **a** and **b** are pooled from three independent experiments and ‘*n*’ indicates total number of individual BMMs analyzed. Influx of sytox blue into macrophages was set to time 0 : 00 to align traces (see ‘Materials and methods’). tdTomato- and GFP-expressing WT macrophages were imaged in the presence of non-fluorescent caspase 1/11-deficient macrophages (please refer to Supplementary Videos 1 and 2). Lines represent the population mean and the shaded areas the 95% confidence interval. (**c**) Corresponding LDH release from imaging experiments in **a** and **b **measured 90 min post-stimulation with RodTox, depicted as mean % LDH release from 1% Triton X-100-treated unstimulated BMMs in each experiment. LDH release measurements in RodTox±5 mM glycine and RodTox *versus* unstimulated are significantly different (two-tailed Student’s *t*-test, *P*=0.0138, *P*=0.00744, respectively). ‘−Gly’, no supplemental glycine; ‘+Gly’, 5 mM supplemental glycine.

The standard LDH assay for cell lysis involves bulk measurement of enzyme release from a population of cells and lacks single-cell resolution. To examine the relationship between cell lysis and cell viability more precisely, we developed a fluorescence microscopy and data analysis workflow to track the kinetics of individual cells as they undergo pyroptosis and lysis. We imaged 1 : 1 : 1 mixtures of wild-type tdTomato-expressing BMMs, wild-type GFP-expressing BMMs, and non-fluorescent caspase-1/11-deficient BMMs stimulated with RodTox±5 mM glycine. To visualize cell membrane integrity and GSDMD pore formation during pyroptosis, we added the dye Sytox Blue, which is excluded from cells with intact plasma membranes.^21^ We processed imaging data to extract time series of fluorescent and morphological parameters for each cell in the imaging field. To compare cells to each other, we aligned the time series of each cell by setting the influx of Sytox Blue to time index 0 : 00 (‘Materials and Methods’). Caspase-1/11-deficient BMMs did not undergo pyroptosis in response to RodTox and served as an internal control to ensure the specificity of the effect of RodTox on cells with an intact inflammasome pathway (data not shown). Following treatment of BMMs with RodTox, we observed a rapid loss of tdTomato fluorescence intensity in wild-type BMMs that begins 4–5 min after Sytox Blue first enters the cell (Figure 2a; Supplementary Video 1). In the presence of 5 mM glycine, we observed that RodTox induced loss of tdTomato fluorophore intensity starting, as before, 4–5 min after Sytox Blue influx. However, tdTomato loss exhibited significantly slower kinetics compared to conditions with no supplemental glycine (rANOVA, *P*=0.00391, Figure 2a; Supplementary Video 2). We also observed a precipitous loss of GFP fluorescence that begins 4–5 min following influx of Sytox blue in wild-type GFP-expressing BMMs treated with RodTox under normal tissue culture conditions (Figure 2b; Supplementary Video 1). Similar to what we observed imaging tdTomato-expressing BMMs, the addition of 5 mM glycine significantly slowed the loss of GFP from GFP-expressing BMMs treated with RodTox (rANOVA, *P*=0.0442, Figure 2b; Supplementary Video 2). The loss of tdTomato and GFP fluorescence in these conditions was not due to photobleaching, as unstimulated BMMs incubated with and without supplemental glycine did not exhibit loss of fluorescent signal comparable to what we observed during RodTox stimulation (data not shown). The loss of cytosolic tdTomato and GFP when lysis is suppressed by glycine suggests that these proteins can escape through GSDMD pores. tdTomato is 6–7 nm in diameter and GFP is 4–5 nm in diameter, both potentially small enough to fit through a GSDMD pore (10–15 nm).^7,8,22^ In fact, we observed that GFP loss proceeds more rapidly than tdTomato loss in the presence of glycine, as would be expected if fluorophore loss is mediated by diffusion through GSDMD pores (Supplementary Figure S2).

Taken together, the above results suggest that RodTox-induced caspase-1 activation is accompanied by loss of lower molecular weight cytosolic proteins (for example, GFP and tdTomato) even in the absence of overt cellular lysis and the loss of higher molecular weight proteins (for example, LDH). To confirm that delayed loss of cytosolic fluorophores in the presence of glycine was associated with retention of LDH we quantified LDH released during imaging experiments. As before, glycine suppressed the release of LDH in cells treated with RodTox (Figure 2c). The larger size of LDH (8–10 nm in diameter)^23,24^ may explain why it is retained within pyroptotic cells when lysis is suppressed by glycine, whereas smaller proteins such as tdTomato and GFP can escape through GSDMD pores. Our results indicate that under normal cell culture and imaging conditions, overt lysis occurs several minutes after GSDMD pore formation. However, if lysis is suppressed, cells retain large proteins (for example, LDH) despite the formation of GSDMD pores and the loss of smaller proteins (for example, GFP or tdTomato).

### Nucleic acid-binding dyes enter pyroptotic cells with variable kinetics according to their size

**Figure 3:**
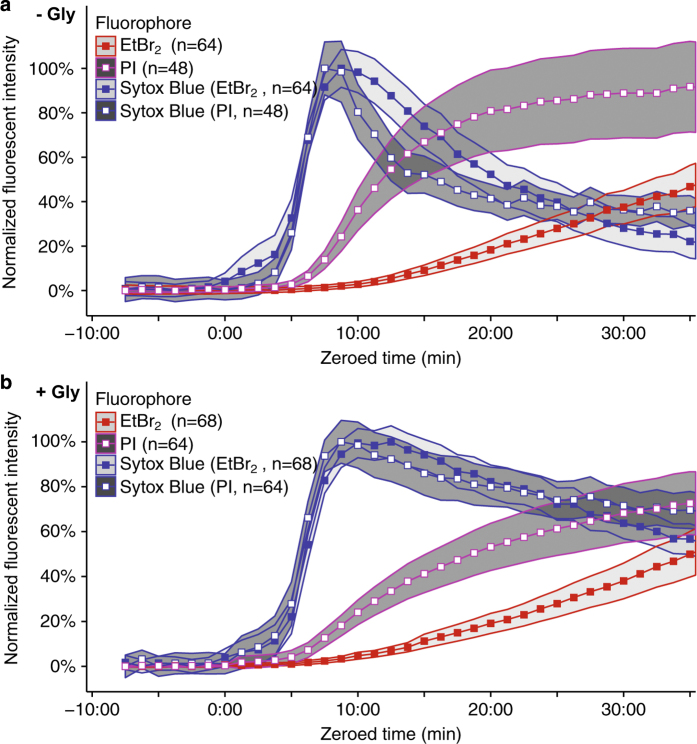
Small-molecular-weight nucleic acid-binding dyes stain pyroptotic BMMs with differential kinetics according to their size. (**a**) Fluorescent intensities over time of Sytox Blue, PI, and EtBr_2_ in non-fluorescent wild-type BMMs stimulated with RodTox in the absence of supplemental glycine. PI and EtBr_2_ staining is significantly delayed relative to Sytox Blue, *P*=0.022 and *P*=0.040, respectively, rANOVA. EtBr_2_ staining is significantly delayed relative to PI staining, *P*=2.9E-12, rANOVA. (**b**) Fluorescent intensities over time of Sytox Blue, PI, and EtBr_2_ in non-fluorescent wild-type BMMs stimulated with RodTox in the presence of 5 mM glycine. PI and EtBr_2_ staining is significantly delayed relative to Sytox Blue, *P*=0.00011 and *P*=2.94E-16, respectively, rANOVA. EtBr_2_ staining is significantly delayed relative to PI staining, *P*=7.76E-9, rANOVA. The delay in PI and EtBr_2_ staining relative to Sytox Blue staining is not significantly distinct±5 mM glycine, *P*=0.097 and *P*=0.394, respectively, rANOVA. In both **a** and **b**, Sytox Blue was paired with either PI or EtBr_2_ in separate imaging wells run in parallel and imaging data were aligned setting influx of Sytox blue to time 0 : 00 (see ‘Materials and Methods’). Lines represent the population mean and the shaded areas the 95% confidence interval. Data in **a** and **b** are pooled from two independent experiments and ‘n’ indicates total number of individual BMMs analyzed. ‘−Gly’, no supplemental glycine; ‘+Gly’, 5 mM supplemental glycine.

Our observation that the smaller protein, GFP, is lost more rapidly than the larger tdTomato from lysis-suppressed, pyroptotic macrophages suggests that molecules small enough to pass through GSDMD pores in pyroptotic cells will do so at different rates according to their size. To test this further, we compared the kinetics of Sytox Blue (400 Da), propidium iodide (PI, 668 Da), and ethidium bromide homodimer (EtBr_2_, 857 Da) by time-lapse imaging as they entered and stained non-fluorescent, wild-type BMMs following treatment with RodTox±5 mM glycine. In the absence of supplemental glycine, we observed differential staining kinetics of PI and EtBr_2_ *versus* Sytox Blue, with each sequentially larger dye staining pyroptotic BMMs more slowly relative to the smallest dye, Sytox Blue (Figure 3a). These results are congruent with a recent study by Russo *et al.*,^9^ which found that PI stains pyroptotic cells faster than EtBr_2_. Also in agreement with Russo *et al.*,^9^ we observed that suppression of BMM lysis with glycine did not significantly delay the influx of PI or EtBr_2_ relative to Sytox Blue (*P*=0.097 and *P*=0.394, respectively, rANOVA comparing the rate of staining by PI and EtBr_2_ relative to Sytox Blue staining±5 mM glycine, Figure 3b). These data suggest that the delayed staining kinetics of larger *versus* smaller molecular weight dyes following inflammasome activation occurs independent of cell lysis and may be regulated by size constraints relative to the size of GSDMD pores in the plasma membrane, although other variables such as dye charge or DNA binding efficiency could also contribute.

### Inflammasome-mediated cell death occurs immediately following membrane permeability even in the absence of cell lysis

**Figure 4:**
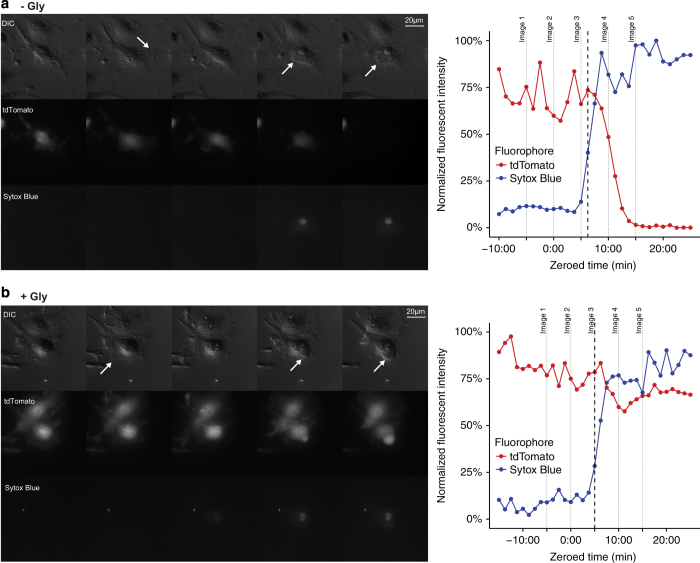
Cells cease ruffling and begin to swell following pore formation even in the absence of lysis. DIC, TRITC (tdTomato), and DAPI (Sytox Blue) channel images taken from imaging time series (left) and quantified fluorescent signal (right) of individual tdTomato-expressing BMMs exposed to (**a**) RodTox or (**b**) RodTox+5 mM glycine, taken at 75 s intervals. Cell movement (in the form of membrane ‘ruffling’) is indicated by an arrow in the second frame. Cell swelling is indicated by arrows in frames 4 and 5. The cessation of cell movement (see ‘Materials and Methods’) is annotated with a dashed line in each graph. Images in **a** and **b** are stills taken from kinetic imaging experiments depicted in Supplementary Videos 3 and 4, respectively, and correspond to the dotted lines labeled ‘Image 1’, ‘Image 2’, etc. in the associated graphs. The data presented are representative of individual macrophages observed in three independent experiments. ‘−Gly’, no supplemental glycine; ‘+Gly’, 5 mM supplemental glycine.

To determine when cell death occurs during pyroptosis, we monitored cell morphology and assessed mitochondrial activity in BMMs treated with RodTox by time-lapse imaging. With or without supplemental glycine, we observed that cell movement ceases immediately following influx of Sytox Blue (Figure 4, and Supplementary Videos 3 and 4). In addition, following Sytox Blue influx, we observed that BMMs proceeded to swell in both normal media and media supplemented with 5 mM glycine (Figure 4, and Supplementary Videos 3 and 4). Swelling is a common feature of pyroptotic cell death that is likely due to GSDMD pore formation in the plasma membrane.^19,20,25^

**Figure 5:**
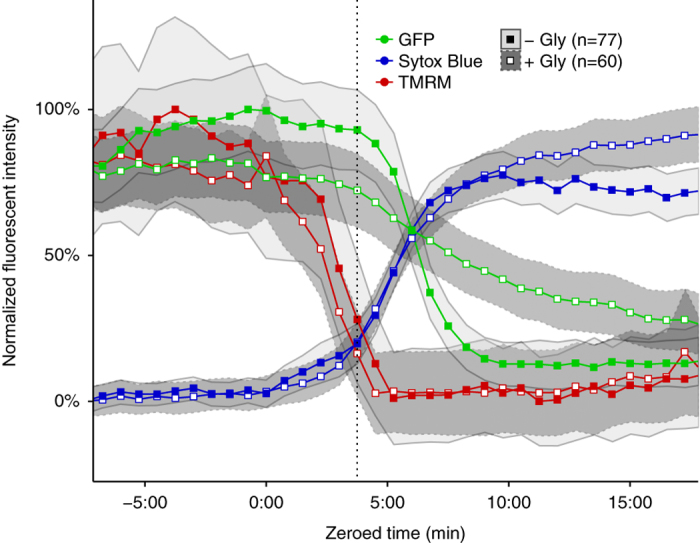
Mitochondrial activity ceases following GSDMD pore formation. Fluorescent intensities of Sytox Blue, GFP, and tetramethylrhodamine methyl ester (TMRM) in GFP-expressing BMMs following exposure to RodTox±5 mM glycine. The start of GFP loss in the no-glycine condition is indicated with a vertical dotted line. Loss of GFP and TMRM fluorescence differ significantly±5 mM glycine (rANOVA, *P*<2E-16, *P*=0.0337, respectively). Data are pooled from six independent experiments and ‘*n*’ indicates total number of individual BMMs analyzed, with cells imaged every 45 s. Lines represent the population mean and shaded areas the 95% confidence interval. Influx of Sytox Blue into macrophages was set to time 0 : 00 to align traces. ‘−Gly’, no supplemental glycine; ‘+Gly’, 5 mM supplemental glycine.

Although a previous study demonstrated that pyroptotic BMMs retain gross intracellular organelle structures such as vacuolar membranes, mitochondria, and the nucleus, it remains unclear when organelle function ceases during pyroptosis.^15^ To measure mitochondrial activity, we imaged BMMs incubated with the cell-permeant dye tetramethylrhodamine methyl ester (TMRM), which sequesters in mitochondria in proportion to mitochondrial membrane potential.^26^ We stimulated a 1 : 1 mixture of wild-type GFP-expressing BMMs and non-fluorescent caspase-1/11-deficient BMMs with RodTox±5 mM glycine. In wild-type BMMs treated with RodTox, TMRM fluorescence intensity began dropping within one minute of Sytox Blue influx regardless of the presence of supplemental glycine, indicating a loss of mitochondrial activity in all pyroptotic cells independent of cell lysis (Figure 5). We did not observe a spike in TMRM fluorescence prior to cell lysis, as was reported in a time-lapse imaging study of cells undergoing necroptosis.^27^ In sum, our cell morphology and mitochondrial activity results suggest that cell death is coincident with GSDMD-mediated cell permeability and that inhibiting cell lysis does not preserve cell viability following pyroptotic stimulus.

### Gasdermin D is required for pore-mediated cell death and cell lysis following inflammasome activation in macrophages

**Figure 6:**
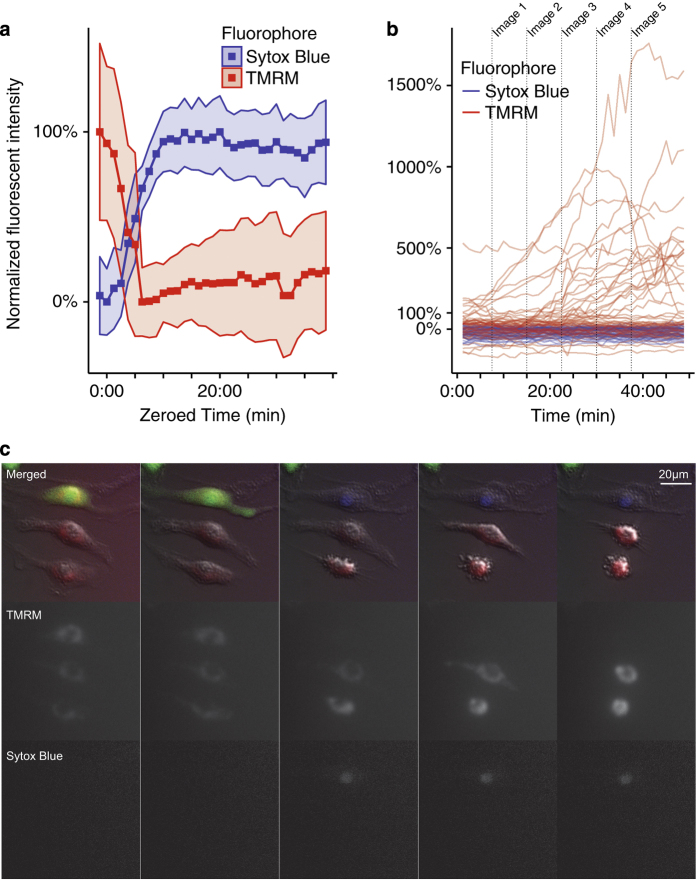
GSDMD is required for loss of cell movement and mitochondrial activity following inflammasome stimulus. (**a**) TMRM and Sytox Blue fluorescence observed in wild-type GFP-expressing BMMs following exposure to RodTox, with traces aligned according to Sytox Blue influx. (**b**) TMRM and Sytox Blue fluorescence observed in non-fluorescent GSDMD-deficient BMMs following exposure to RodTox. Traces from each cell were not aligned and instead are depicted over the timecourse of the experiment. TMRM fluorescence intensity in GSDMD-deficient BMMs is scaled relative to wild-type GFP-expressing controls such that 100% fluorescence intensity of GSDMD-deficient BMMs is equivalent to the mean 100% fluorescence intensity of wild-type controls (see Materials and methods). (**c**) Time series of TMRM, Sytox Blue, and merged-channel (showing DIC, GFP, TMRM, and Sytox Blue) images wild-type GFP-expressing and non-fluorescent GSDMD-deficient BMMs following exposure to RodTox in the presence of TMRM. Following pretreatment with TMRM, WT and GSDMD-deficient BMMs were stimulated with RodTox and imaged in the same wells (please refer to Supplementary Video 5). Images in **c** are stills taken from a kinetic imaging experiment depicted in Supplementary Video 5, and correspond to the dotted lines labeled ‘Image 1’, ‘Image 2’, etc. in **b**. Data depicted are representative of three independent experiments.

To determine whether GSDMD is required for the morphological and physiological events associated with cell death and cell lysis following inflammasome activation in individual BMMs characterized above, we stimulated 1 : 1 mixtures of wild-type GFP-expressing BMMs and non-fluorescent BMMs derived from *Gsdmd*^*−/−*^ mice^28^ with RodTox in the presence of Sytox Blue and TMRM. Whereas wild-type GFP-expressing BMMs behaved as characterized in Figure 5, following stimulation with RodTox, we did not observe GSDMD-deficient BMMs become permeable to Sytox Blue or lose mitochondrial activity as measured by TMRM fluorescence (Figures 6a and b; Supplementary Video 5). In fact, we observed that in 41% of GSDMD-deficient BMMs, RodTox treatment induced morphological changes associated with apoptosis, including cellular rounding, shrinking, and bleb formation (Figure 6c; Supplementary Video 5). We observed a transient increase in TMRM fluorescence in GSDMD-deficient BMMs displaying these morphological changes (Figures 6b and c, and Supplementary Video 5). This increased TMRM fluorescence could result from reorganization of mitochondria or altered mitochondrial activity following inflammasome activation in the absence of GSDMD. GSDMD-deficient BMMs responded to RodTox identically in 5 mM supplemental glycine, indicating that suppression of cell lysis had no effect on the effects of RodTox on these cells (data not show). These observations suggest that GSDMD-deficient BMMs can undergo apoptosis following exposure to RodTox. Apoptotic cell death following caspase-1 activation in the absence of GSDMD has been described in mouse and human macrophage cell lines and may contribute to cell death observed in other cell types in the absence of non-functional GSDMD following inflammasome activation *in vivo*.^28,29^ Importantly, our results demonstrate that GSDMD is required for the membrane permeability, loss of cell movement, and loss of mitochondrial activity that we associated with cell death following pyroptotic stimulus.

## Discussion

In the context of normal tissue culture conditions, inflammasome activation in BMMs results in overt cell lysis and the loss of large cytosolic proteins such as LDH. However, a growing body of evidence suggests that other cell types, including neutrophils, fibroblasts, and human monocytes, can experience inflammasome activation in the absence of lysis.^11–14^ In addition, prior studies have found that the anti-lytic reagent glycine can block inflammasome-induced cell lysis, measured both by LDH release and the loss of fluorescent proteins from the cell cytosol.^17–20^ Although inhibiting cell lysis has not been found to disrupt upstream pyroptotic events, such as the processing and release of IL-1*β* and IL-18, blocking lysis has been interpreted as the equivalent of blocking cell death.^18,30,31^ Though there are no absolute diagnostic criteria for determining whether a cell is alive or dead, we observed that cell movement and mitochondrial activity ceased coincident with plasma membrane permeability caused by the formation of GSDMD pores, whether or not the cell proceeded to lyse (Figures 4 and 5). Likewise, we observed that an equivalent proportion of BMMs ultimately lysed following treatment with RodTox regardless of whether we initially inhibited lysis with glycine (Figure 1). These results strongly suggest that pyroptotic cell death can be uncoupled from cell lysis, and that preventing lysis does not prevent cell death following pyroptotic stimulus.

Notably, we did not observe that any parameters associated with pyroptosis occurred prior to the formation of GSDMD pores in the plasma membrane, as measured by uptake of Sytox Blue. This suggests that pyroptotic cell death does not occur prior to GSDMD pore formation in the plasma membrane, in line with prior studies.^7,8^ Other work has shown that pyroptosis results in degraded nuclear DNA via an ICAD-independent mechanism and that activated caspase-1 degrades glycolytic enzymes.^19,32^ Whether these events contribute to cell pathology or cell death in parallel with the activity of GSDMD pores remains to be determined.

Even in the absence of supplemental glycine, we found that cell lysis of macrophages is delayed relative to cell death. Using time-lapse fluorescent microscopy, we found that once a cell becomes permeable to Sytox Blue, a marker for GSDMD pore formation, cell movement stops. In addition, mitochondrial membrane potential dissipates and the cell begins to swell. However, cytosolic fluorophores do not begin to dissipate until 4–5 min after membrane permeabilization, at which point they dissipate rapidly (Figure 2). Inhibition of cell lysis with glycine does not delay the initiation of cytosolic fluorophore loss, but does considerably slow the rate of cytosolic fluorophore loss. These data suggest that glycine does not prevent cell death or leakage of lower molecular weight cellular contents during pyroptosis but instead maintains the integrity of the plasma membrane of an otherwise dead cell. Furthermore, we observed that nucleic acid-binding dyes leak into pyroptotic BMMs at rates according to their size (Figure 3). This observation may explain why a previous study observed differential kinetics of pyroptotic cell staining comparing EtBr and EtBr_2_.^19^

Our results are distinct from those of Estacion *et al.*^20^ investigating pyroptosis in bovine vascular epithelial cells treated with maitotoxin, which demonstrated that inhibition of lysis appeared to preserve cell viability as cells treated with 5 mM glycine and maitotoxin for four hours or longer excluded ethidium bromide. In our experiments, BMMs treated with RodTox for five hours did not exclude ethidium bromide, whether or not they were treated with 5 mM glycine (Supplementary Figure S1). This discrepancy may be due to differences in how maitotoxin and RodTox trigger pyroptosis or differences in pyroptosis of bovine vascular endothelial cells *versus* murine BMMs.

Though the mechanism by which glycine protects cells remains mysterious, our data suggest pyroptosis does not involve a ‘glycine-sensitive death channel,’ as has been suggested.^10^ Rather, we propose that GSDMD oligomers create pores that collapse the electrochemical gradient of the cell, effectively killing it. In tissue culture, resulting water influx raises intracellular pressure, tearing the cell plasma membrane and releasing large molecules such as LDH. Under this model, inhibiting cell lysis does not prevent cell death. Thus, we consider pyroptosis to be ‘gasdermin-dependent cell death’. This definition is similar to a recent proposal by Shi *et al.*^6^ suggesting that pyroptosis be defined as ‘gasdermin-dependent programmed necrosis’ but differs in the assumptions made about the lytic nature of pyroptosis. Our study suggests that overt cell lysis can result from pyroptosis but is not a necessary consequence of pyroptosis. Therefore, we propose that cell death and cell lysis should be considered distinct events in the context of pyroptosis.

The ability to uncouple cell death from cell lysis raises the possibility that pyroptosis may not always result in cell lysis *in vivo*. Pyroptotic cell death in the absence of cell lysis could occur without releasing large cytosolic components into surrounding tissue. Our data are therefore congruent with a recent proposal by Jorgensen *et al.* that one of the functions of pyroptosis is to trap intracellular pathogens within the cell carcass, facilitating uptake by immune cells and pathogen clearance.^15^ However, although Jorgensen *et al.* propose that PIT formation is a consequence of cell lysis, our data suggest that cell lysis may not be required for cell death in response to intracellular bacteria. Several other cell types (discussed above) that have previously been suggested not to undergo lysis during pyroptosis still might die, for example, human monocytes, neutrophils, and fibroblasts. Our data suggest that pyroptotic cell death without lysis still allows for dissipation of cytosolic molecules small enough to diffuse through GSDMD pores. This could facilitate the escape of cellular metabolites, the absence of which would prevent intracellular pathogens from taking advantage of the intracellular niche of a pyroptotic cell. In fact, Russo *et al.*^9^ observed that ATP loss from pyroptotic macrophages did not require cell lysis. It remains to be determined whether cell death is uncoupled from cell lysis during exposure to pyroptotic stimuli *in vivo*, and whether pyroptosis in the absence of lysis allows for better control of intracellular pathogens and inflammation.

## Materials and methods

### Ethics statement

We conducted this study in accordance with guidelines contained in the *Guide for the Care and Use of Laboratory Animals* of the National Institutes of Health and under a protocol approved by the Animal Care and Use Committee at the University of California, Berkeley (protocol number AUP-2014-09-6665).

### Derivation of bone marrow-derived macrophages

We derived macrophages from mouse bone marrow (BMMs) in RPMI 1640 media supplemented with 10% fetal bovine serum, 2 mM l-glutamine, 100 μM streptomycin (all from ThermoFisher Scientific, Waltham, MA, USA), and 5% supernatant from 3T3 cells expressing macrophage colony-stimulating factor (generated in-house). We derived non-fluorescent wild-type BMMs from C57BL/6J mice (Jackson Laboratories), non-fluorescent GSDMD-deficient BMMs from *Gsdmd*^*−/−*^mice on the C57BL/6J background (described previously^28^), and non-fluorescent caspase 1/11-deficient BMMs from *Casp1/11*^*−/−*^ mice on the C57BL/6J background^33^ (a gift from A van der Velden and M Starnbach at Harvard Medical School). We derived tdTomato-expressing BMMs from C57BL/6J.SJL mice expressing floxed-stop tdTomato in the Rosa locus that were crossed to E2a-Cre-expressing C57BL/6J mice to excise the stop cassette and then crossed again to remove the presence of Cre (provided by the laboratory of Gregory Barton at U.C. Berkeley). We derived GFP-expressing BMMs from C57BL/6J mice expressing a floxed stop-GFP cassette ahead of a toll-like receptor 9-HA transgene (provided by the laboratory of Mark Schlomchik at the University of Pittsburgh). BMMs derived from these mice lack Cre, express ubiquitous GFP, and do not express the transgene.

### RodTox

We generated recombinant protective antigen (PA) from *B. anthracis* and recombinant inner rod protein PrgJ from the *S.* *typhimurium* SPI-1 type III secretion system fused to the N-terminal domain of anthrax lethal factor (LFn-PrgJ) to generate RodTox, as previously described.^16^

### Pyroptosis and LDH release assay

We plated 100 k bone marrow-derived murine macrophages (BMMs) overnight in 100 *μ*l per well of complete RPMI (RPMI 1640 media+10% fetal bovine serum, 2 mM l-glutamine, 25 mM HEPES, and 100 *μ*g/ml streptomycin (all from ThermoFisher Scientific)+5% macrophage colony-stimulating factor-enriched media). On the day of the assay, BMMs were treated with 100 *μ*l of one of six possible conditions: RodTox (32 ng/ml LFn-PrgJ+4 *μ*g/ml PA), RodTox*+*5 mM glycine (Sigma, St Louis, MO, USA), 5 mM glycine alone, media alone, or 1% Triton X-100 (Sigma). We incubated BMMs in these conditions at 37 °C+5% CO_2_ for 90 min and sampled 50 *μ*l supernatant from each well and visualized LDH release as described previously.^34^ For sequential LDH release analysis, we stimulated cells at 37 °C+5% CO_2_ for 90 min, after which we sampled 50 *μ*l supernatant from each well. We then washed the cells 2× with warm PBS and replaced media with 100 *μ*l of either media alone, media+5 mM glycine, or 1% Triton X-100. After incubating for 30 min at 37 °C with 5% CO_2_, we sampled 50 *μ*l of supernatant from each well. We then replaced media with 100 *μ*l/well of a 1% Triton X-100 solution in PBS. After waiting 5 min, we sampled 50 *μ*l of supernatant from each well. LDH release was normalized to percent of LDH signal from BMMs treated with 1% Triton X-100 after correcting for background LDH signal in media-alone conditions.

### Imaging

We plated 50 k cells per well in complete RPMI lacking phenol red (ThermoFisher Scientific) in Nunc Lab-Tek II 8-well chamber slides (ThermoFisher Scientific #154534). We recorded time-lapse videos using NIS Elements II and an inverted live-cell microscope (Nikon, Melville, NY, USA) equipped with a cell incubation chamber that provided CO_2_, heating, and humidity control. To visualize cell permeability, we added 0.5 *μ*M Sytox Blue to the media. For each experiment, we tested five conditions concurrently: RodTox alone, RodTox+5 mM glycine, 5 mM glycine alone, media alone, and 1% Triton X-100. Following imaging, we sampled 50 *μ*l of supernatant from each well to perform an LDH release assay as described above. For imaging of mitochondrial activity, we treated cells with 200 ng/ml of tetramethylrhodamine methyl ester perchlorate (TMRM, Life Technologies, Carlsbad, CA, USA) for 30 min before replacing with stimulation conditions. For nucleic acid dye staining kinetics experiments, we added 1 μM Sytox Blue, 1 μM propidium iodide (Sigma), and 1 μM ethidium bromide homodimer (Life Technologies) as indicated.

### Data extraction

We used Imaris 8 (Bitplane, Concord, MA, USA) to computationally identify each cell in each frame of collected images using cell fluorescence or DIC contrast to mark the cell surface, using background subtraction and surface splitting. This produced multiple data sets for each identified cell: one for DIC and one per each fluorescence detection channel. We combined data sets into a composite set by constructing a 25 *μ*m×25 *μ*m bounding box around each identified cell and grouping any other identified cells whose centers were contained by the bounding box ⩾75% of the time. To reduce transient noise, we removed any group members that existed for fewer than 11 frames and any groups that existed for ⩽25 frames.

### Determination of cell membrane permeability

We programmatically determined if and when cells became permeable using Sytox Blue fluorescent intensity. We first scaled Sytox Blue intensities between the minimum and maximum observed intensity in each experimental condition. After generating a continuous variable using a cubic smoothing spline, we constructed a sliding window average of the slope of the curve with a window size of 5 min. The earliest frame in the window in which the slope was ⩾4.0E−4 was marked as the start of Sytox Blue influx. If no window was found in which the slope exceeded this value, we considered the cell to have remained unpermeabilized.

### Cell partitioning

We categorized BMMs as tdTomato-positive, GFP-positive, or non-fluorescent controls based on the fluorophores present in each cell and their intensity. For each fluorophore, we calculated 1 standard deviation above the background mean as the minimum fluorophore threshold per frame. Cells in which fluorophore intensity was below the corresponding threshold over the course of the entire experiment were considered to be fluorophore-negative. Cells positive for Sytox Blue at the beginning of the experiment, or which appeared on visual inspection to be misclassified were excluded from analysis.

### Trace alignment and fluorophore intensity normalization

Fluorophore intensity was normalized per fluorophore, per experiment against the minimum and maximum observed fluorescence intensity across all experimental conditions in a single experiment. We combined normalized data from multiple experiments to generate the figures in this study. Traces for each composite surface were translated along the time axis such that the calculated start of Sytox Blue influx as described above was set to 0 : 00, and graphs were truncated to remove regions where fewer than 65% of the composite traces were represented. Finally, the mean values of each fluorophore were scaled to lie from 0 to 100%. For GSDMD-deficient BMMs, the TMRM values for each cell were scaled against the maximum and minimum mean population values of control wild-type GFP-expressing cells.

### Determination of the start of cytosolic fluorophore loss and assessment of cell movement/morphology

We determined the start of tdTomato or GFP loss and PI and EtBr_2_ influx using the *pastecs* R package (https://cran.r-project.org/package=pastecs) to identify the turning points in the mean values for each fluorophore. We assessed BMM movement by visually inspecting kinetic imaging series frame by frame to observe variables such as directional movement within the imaging plane, extension and retraction of pseudopodia, and apparent membrane ruffling. We defined cessation of movement for any given cell as the frame in which none of these parameters were visually apparent. We also visually assessed cell swelling as rounded membrane extrusions that enlarged from BMMs following dye influx and cessation of cell movement.

## Additional information

**Publisher’s note:** Springer Nature remains neutral with regard to jurisdictional claims in published maps and institutional affiliations.
