# Supplementary figures and images for: Cell death and cell lysis are separable events during pyroptosis
Source: Cell Death Discov. 2017 Nov 13;3:17070–. doi: 10.1038/cddiscovery.2017.70 (PMC5682879; doi:10.1038/cddiscovery.2017.70)

**Fig. S1**

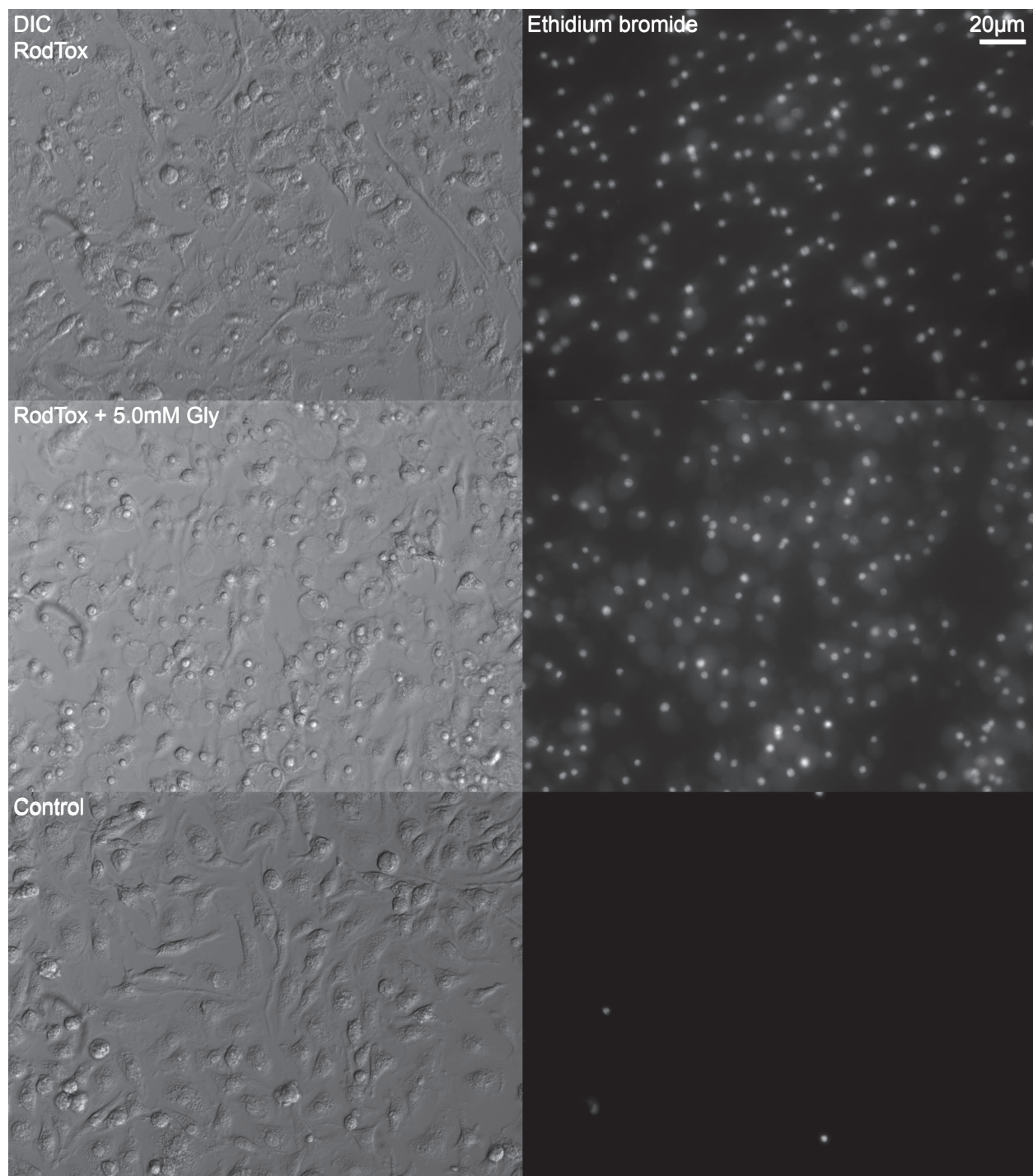

Supplement: Supplementary Figure S1 [file cddiscovery201770-s1.pdf]

**Fig. S2**

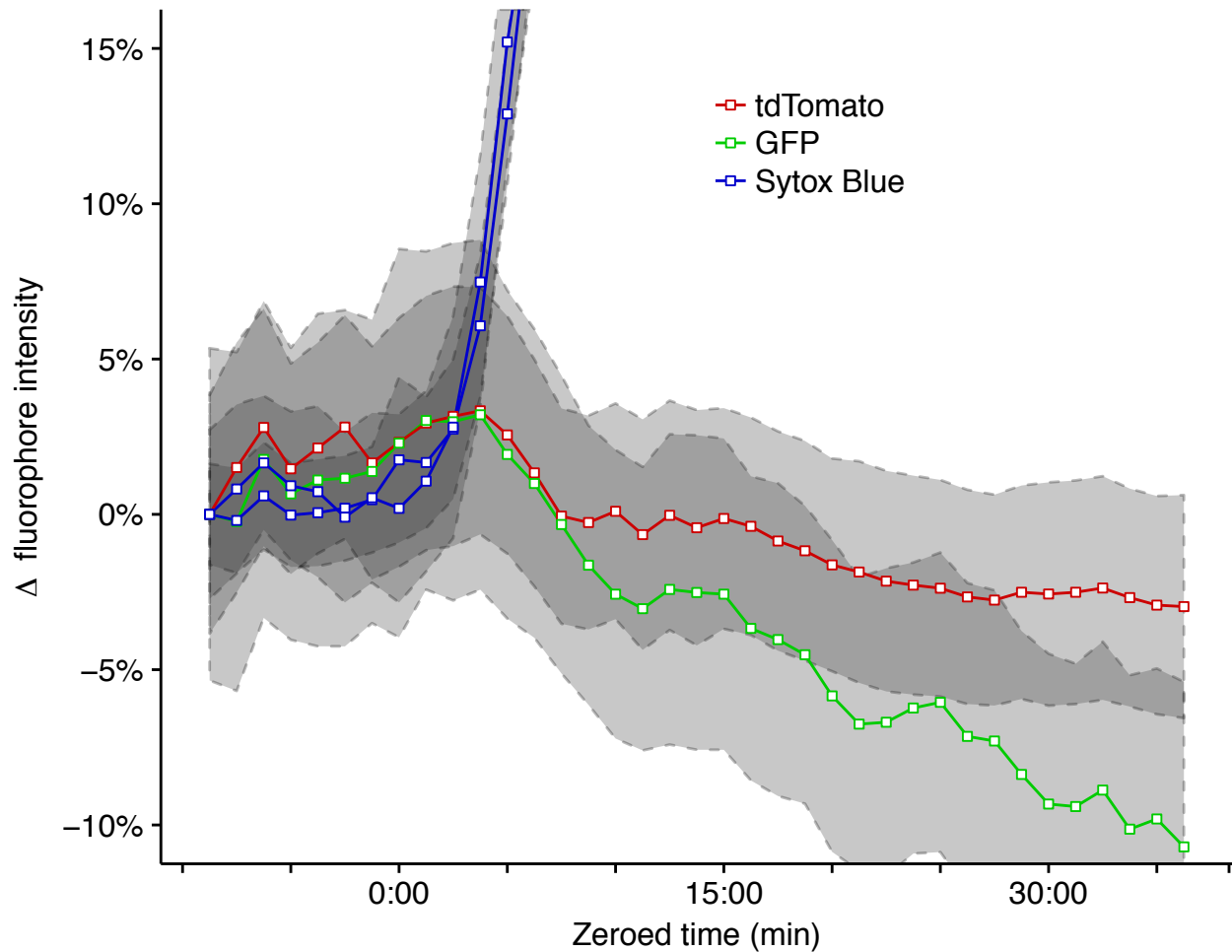

Supplement: Supplementary Figure S2 [file cddiscovery201770-s2.pdf]
